# Human iPSC-Derived Spinal Cord Organoids – The First Electrophysiological Assessment of Neuronal Maturation

**DOI:** 10.1021/acschemneuro.5c00823

**Published:** 2026-07-28

**Authors:** Oleksandr Ievglevskyi, Denis Zosen, Jordi Requena Osete, Joel C. Glover, Ragnhild Elisabeth Paulsen, Srdjan Djurovic, Elena Kondratskaya

**Affiliations:** † Department of Medical Genetics, 155272 Oslo University Hospital, Oslo 0450, Norway; ‡ Neurobiology, SISSA, Trieste 34136, Italy; § Computational and System Neuroscience, ISAS, Camerino 62032, Italy; ∥ Section for Pharmacology and Pharmaceutical Biosciences, Department of Pharmacy, University of Oslo, Oslo 0371, Norway; ⊥ Centre for Precision Psychiatry, Division of Mental Health and Addiction, University of Oslo, and Oslo University Hospital, Oslo 0450, Norway; # Laboratory of Neural Development and Optical Recording (NDEVOR), Division of Physiology, Department of Molecular Medicine, Institute of Basic Medical Sciences, University of Oslo, Oslo 0372, Norway; ∇ Norwegian Center for Stem Cell Research, Institute of Immunology and Transfusion Medicine, Oslo University Hospital, Oslo 0372, Norway

**Keywords:** human pluripotent stem cells, spinal cord organoids, whole-cell patch clamp, electrophysiological recording, neuronal maturation, development

## Abstract

The spinal cord exhibits a complex cytoarchitecture and neuronal circuitry organization that challenges in vitro replication of its functional integrity. Here we employed a previously published protocol to generate spinal cord organoids (SCOs) from human-induced pluripotent stem cells (hiPSCs). We characterized phenotypic changes in functional neuronal properties in SCO neurons at early developmental stages (15–32 days in culture) using a quantitative electrophysiological approach. Using the whole-cell patch-clamp technique to evaluate electrophysiological properties, we found that SCO neurons exhibited progressive maturation, as evidenced by hyperpolarized resting membrane potentials, increased inward current amplitude, refined action potential kinetics, and the early emergence of mature-type firing patterns. In particular, we show that the spike frequency adaptation phenomenon, which prevails in motor neurons, appears at early stages of SCO neuron development. Immunohistochemical assessment confirmed the expression of key transcription factors in motor neurons (ISLET1 and HB9) and immature spinal interneurons (LHX1/5 and PAX2). Collectively, our findings demonstrate that neurons in hiPSC-derived SCOs exhibit physiological differentiation, which is important for using SCOs to investigate human spinal cord development and advance translational research in CNS disorders and cell replacement therapies.

## Graphical abstract

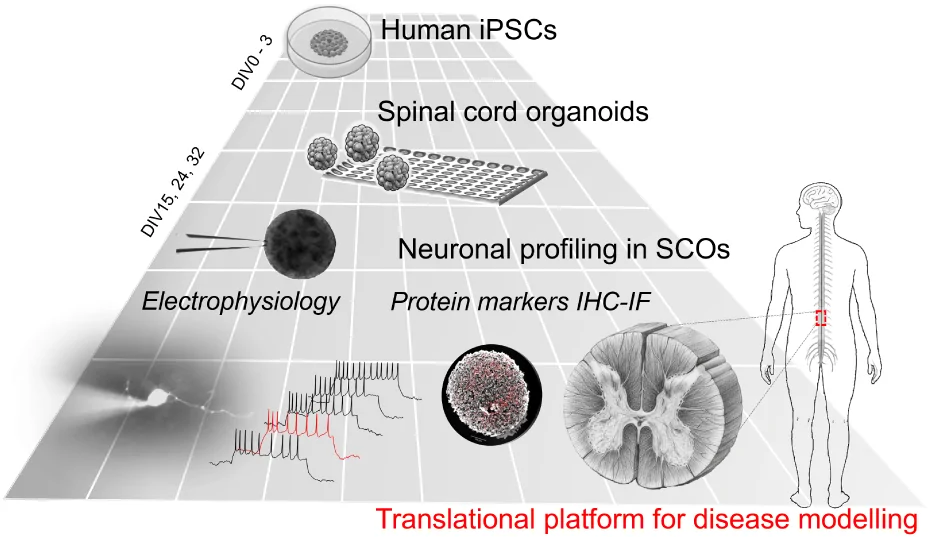

## Introduction

Human-induced pluripotent stem cells (hiPSCs) provide an effective platform for generating human neural tissue for in vitro modeling of human neurophysiology and neurological diseases. By utilizing defined differentiation protocols, it is possible to direct hiPSCs to create self-organized three-dimensional (3D) neural organoids that mimic the structural and molecular properties of specific regions of the central nervous system.^1,2^ In particular, spinal cord organoids (SCOs) hold significant promise for investigating the pathophysiology of diseases such as spinal muscular atrophy (SMA),^3,4^ amyotrophic lateral sclerosis (ALS),^5^ multiple sclerosis (MS), other demyelinating diseases,^6–8^ and spinal cord injury.^9^ SCOs can provide an accurate physiological context for exploring disease mechanisms and potential treatments,^10^ including tissue replacement therapies.

The spinal cord plays a pivotal role in encoding and relaying sensory information to higher brain centers, as well as transmitting descending commands for appropriate motor responses. These complex functions are enabled by its intricate cytoarchitecture and neuronal circuitry organization. During development, the spinal cord establishes three principal axes—the anterior–posterior (AP or rostral–caudal), dorsal–ventral (DV), and medial–lateral (ML) axes.^11^ The formation of the DV axis starts early in neural tube development and is tightly regulated by morphogens: Sonic Hedgehog (Shh) directs ventral patterning, while bone morphogenetic proteins (BMPs) and Wnt signaling contribute to dorsal patterning of the roof plate.^12–16^ Motor neurons generated in the ventral region are clustered into muscle-specific motor neuron pools that are organized in columns and columnelles according to muscle targets; sensory interneurons in the dorsal region exhibit a laminar organization related to sensory modality; and a large variety of additional interneurons are organized into integrative circuits, including central pattern generators for rhythmic movements. Such complexity substantially impedes in vitro generation of 3D spinal cord multineuronal cultures.

The generation of organoids typically involves several steps, including embryoid body formation, neural induction, and tissue maturation, starting from either embryonic stem cells^17,18^ or iPSCs.^19^ Ogura et al.^20^ pioneered the induction of complex human spinal cord tissue, and more recently, self-organizing 3D human spinal cord-neuromuscular organoids have been generated.^21^

Electrophysiological recordings are essential for quantitatively evaluating the functional neuronal activity of any origin. Prior electrophysiological assessment of hiPSC-derived mature SCOs has been done mainly using multielectrode arrays (MEAs)^22,23^ because the more powerful patch-clamp recording method is technically difficult to apply to organoids due to size and accessibility constraints. Consequently, information regarding the early stages of neuronal maturation, transitional changes, and emerging phenotypes in SCOs is sparse.

In this study, we provide the first detailed functional characterization of individual neurons in whole, intact SCOs, highlighting the electrophysiological properties associated with their developmental maturation, as obtained through whole-cell patch-clamp recordings.

## Results

### Characterization of Passive Neuronal Properties in hiPSC-Derived SCOs

Spinal cord organoids (SCOs) were cultured for up to 32 days following a previously established protocol.^20^ Whole-cell patch clamp recordings were performed on neurons from intact organoids with diameters ranging from 200 to 600 μm at days in vitro DIV15, DIV24 and DIV32. Representative fluorescence images of neurons are shown in Figure 1A. Of the 134 recorded neurons, 54 displayed activation of voltage-dependent membrane currents and were selected for further analysis.

**Figure 1:**
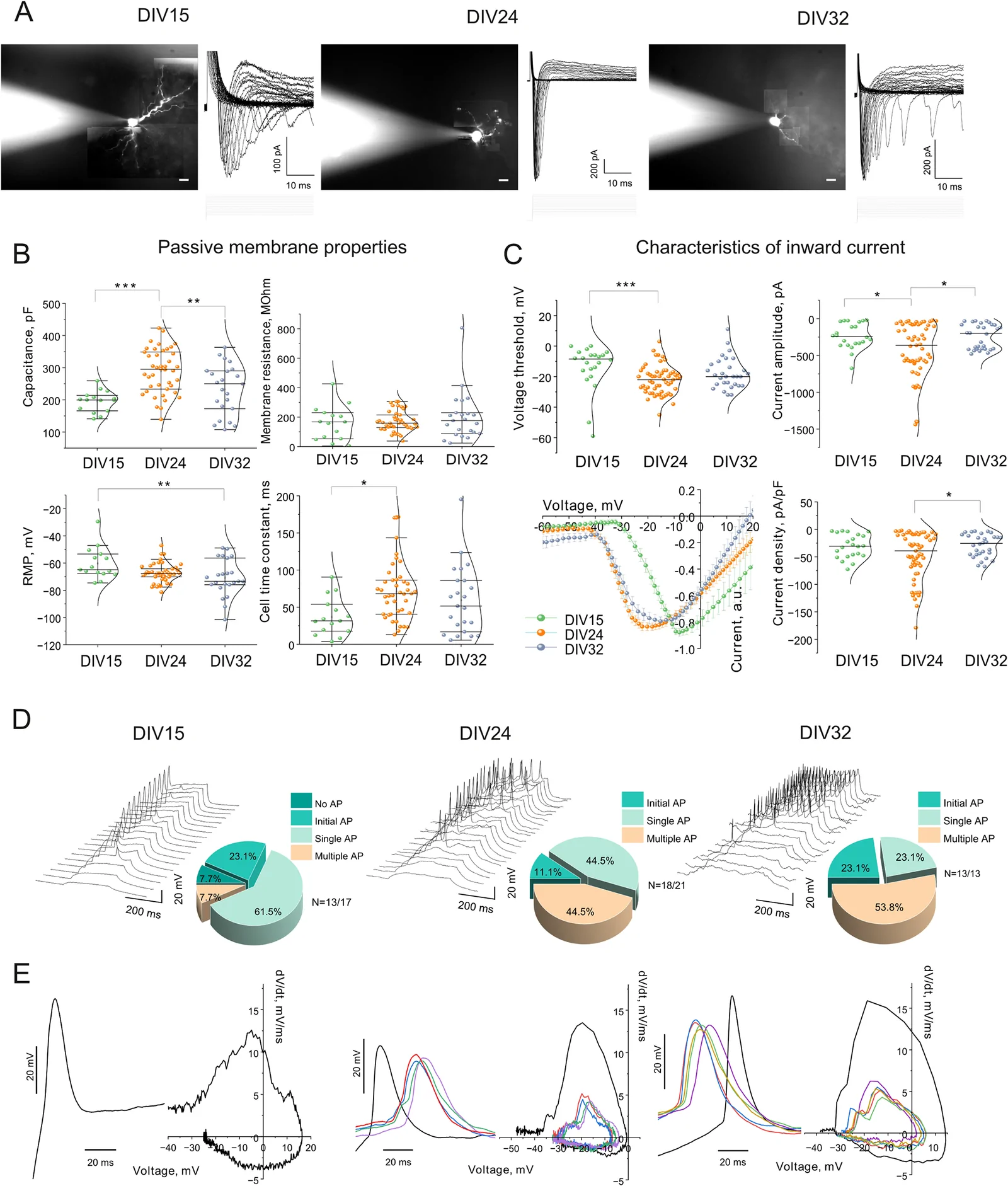
Electrical properties of SCO neurons: membrane properties, whole-cell currents, and evoked action potentials. A, left panels—representative fluorescence images with reconstructed morphology of the recorded neurons from the indicated age groups (Alexa Fluor 488). The right panel shows original traces of transmembrane currents evoked by step depolarization (*V*_hold_ = −60 mV) in the shown cell. Scale bar: 10 μm. B—Passive membrane properties of SCO neurons. Upper panels—Violin plots of whole-cell capacitance values (C, pF) estimated for recorded neurons in the DIV15, DIV24, and DIV32 groups. There was a significant difference between the DIV15 and DIV24 groups (*p* < 0.001, ANOVA and Bonferroni test), as well as between DIV24 and DIV32 (*p* < 0.05). Summary of the membrane resistance values (MΩ) in the examined groups. Lower panels—violin plots representing the rest membrane potentials (RMP, mV) in the DIV15, DIV24, and DIV32 groups. RMP values were significantly lower in the group DIV32 compared to DIV15 (*p* < 0.05). Graph plots for the cell time constant (ms). Values are significantly different (*p* < 0.05) for the DIV15 versus DIV24 comparison. C—Analysis of inward currents in SCO neurons. Upper panels—Violin plots for the voltage threshold, *V*_t_, for inward currents. *V*_t_ values significantly differ between DIV15 and DIV24 (*p* < 0.001) . Data were analyzed by ANOVA and Bonferroni test. Summary of inward current amplitudes, with each dot representing an individual recording. Average amplitude values were significantly increased in DIV24 compared to DIV15 (*p* < 0.05), as well decreased in the group DIV32 compared to DIV24 (*p* < 0.05, ANOVA and Bonferroni test). Lower panels—Current–voltage (I–V) relationship curves for inward currents recorded in neurons in DIV15, DIV24, and DIV32 groups. Summary graph showing the peak current density (pA/pF). Average values were significantly different for DIV24 vs DIV32 (*p* < 0.05). Data were analyzed with One-Way ANOVA followed by Bonferroni posthoc test). D—Representative traces of AP evoked in step current injection protocol at DIV15, DIV24 and DIV32 neurons. Lower panels—Specific AP waveforms recorded in neurons from each group (%).E—Representative AP waveform and phase plane plots (d*V*/d*t*, mV/ms vs V, mV) typical for neurons in each group.

We first analyzed the key passive membrane properties of the recorded neurons (Figure 1B). Membrane capacitance increased significantly on pairwise comparisons from DIV15 to DIV24, and decreased from DIV24 to DIV32 (Figure 1B, Table S1). Membrane resistance was close to 0.2 GΩ and did not differ significantly over time in vitro (Figure 1B, Table S1).

Resting membrane potentials (RMPs) tended to become more negative as SCOs matured and differed significantly in pairwise comparisons of DIV15 versus DIV32 (Figure 1B, Table S1).

The membrane time constant was significantly increased in comparison between DIV15 and DIV24 (Figure 1B).

Additional passive and active membrane properties analyses are shown in Supporting Information Tables S1-S4.

### Transmembrane Currents

Recorded SCO neurons generated robust inward and outward transmembrane currents in response to depolarization. Representative traces of whole-cell currents are shown in Figure 1A, right, and average current–voltage (I–V) curves are shown in Figure 1C.

V_t_ for inward current activation differed between DIV15 and DIV24, as well as between DIV15 and DIV32 (Figure 1C). Inward current amplitudes were lowest at DIV15, increased significantly at DIV24, and then decreased again at DIV32 (Figure 1C). Peak current density was also highest at DIV24, and significantly different in pairwise comparisons of DIV15 versus DIV24 and DIV24 versus DIV32 (Figure 1C, Table S2). Averaged I–V curves normalized to maximum inward current (au vs V) are shown in Figure 1C. IV curves for inward and outward current densities (pA/pF vs V) are shown in Supporting Information Figure S1A.

### Action Potential Properties of Neurons in SCOs

Most recorded neurons at each stage generated single or multiple action potentials (APs) upon depolarizing step current injection in current-clamp mode, as shown in Figure 1D. Thus, neurons were grouped according to their firing profile, with elicited initial type AP, single AP, or multiple APs. As shown in Figure 1D, at DIV15, the majority of neurons fired single APs, whereas about half of the neurons at DIV24 and DIV32 fired multiple APs.

The phase-plotted voltage derivative over time (d*V*/d*t*, vs voltage) was used to determine AP threshold, depolarization, and repolarization slopes (Supporting Information Figure S1B). As shown by representative APs at each stage of maturation (first evoked AP in a series shown in black and subsequent APs in other colors), the AP waveform changed over time in the culture (Figure 1E).

Since the AP waveform reflects the degree of functional maturation,^24,25^ we therefore analyzed individual AP characteristics according to waveform features (Supporting Information, Figure S1B,C), including amplitude, threshold, half-width at half-maximum amplitude (here termed HWHM), AP kinetic parameters, rise and decay time constants (tau), and afterhyperpolarization (AHP) parameters.

AP thresholds did not vary significantly among groups, while AP amplitudes were significantly greater at DIV24 relative to those at DIV15 and to those at DIV32 (Figure 2A).

**Figure 2:**
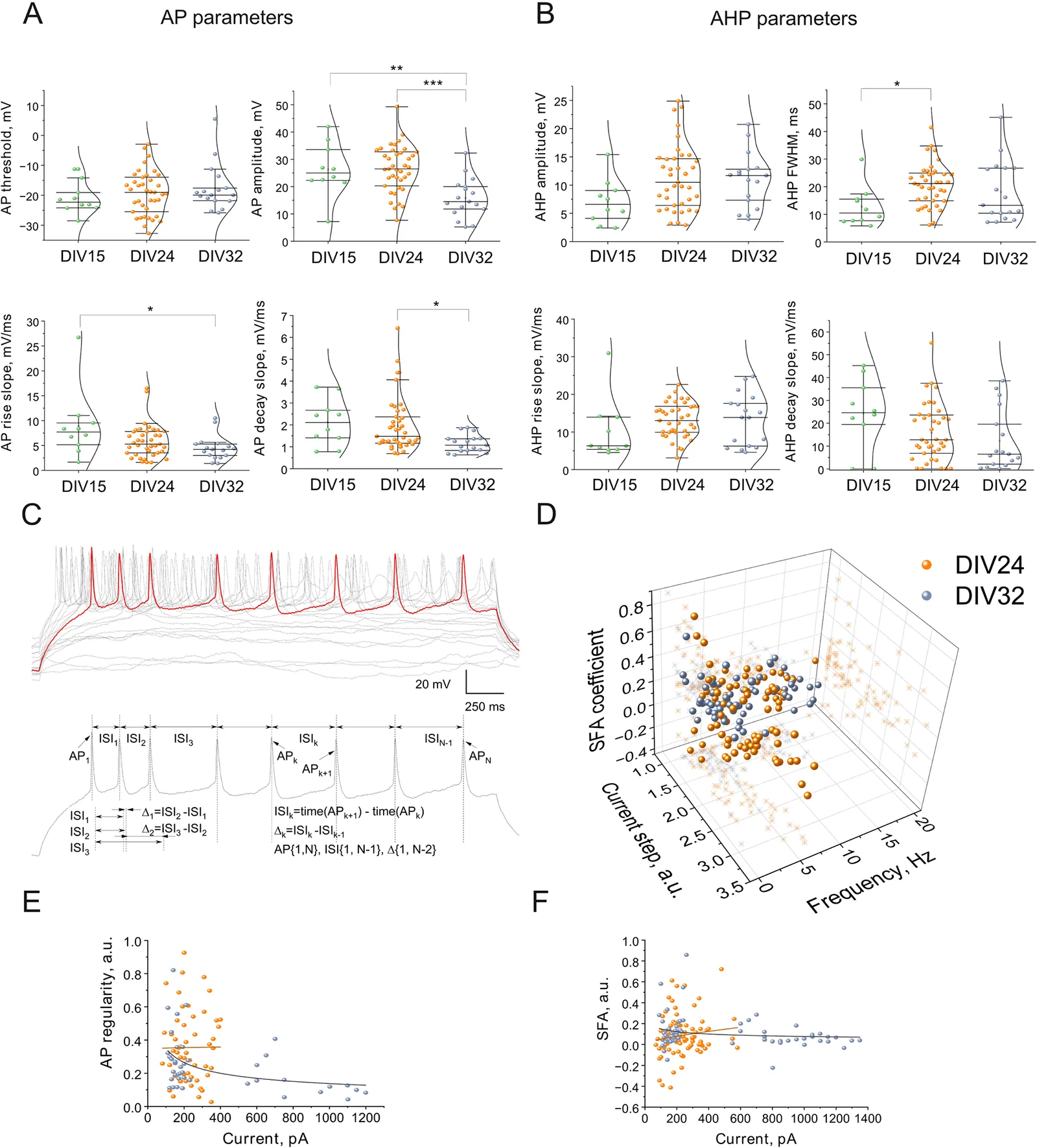
AP, AHP and firing patterns′ parameters A—AP parameters. Upper panels—Summary plot for AP thresholds. Summary of action potential amplitudes (AP amplitude, mV) recorded from neurons in the respective groups. Average AP amplitude values were significantly different between DIV15 and DIV32 (*p* < 0.01) and between DIV24 and DIV32 (*p* < 0.001). Lower panels—Kinetic parameters of APs corresponding to tau rise (left panel) and tau decay (right panel). Tau rise values are significantly different between DIV15 and DIV32 (*p* < 0.05), with decreased AP rise time in DIV32. Tau decay differed significantly in comparison DIV24 vs DIV32 (*p* < 0.05). B—AHP parameters. Upper panels—Summary plot for AHP amplitudes and AHP fwhm. Values of AHP fwhm were significantly different for DIV15 and DIV24 (*p* < 0.05). Lower panels—AHP kinetic parameters (tau rise and tau decay). Data were analyzed with One-Way ANOVA followed by Bonferroni posthoc test). C—Graphical chart for the analysis of the spike frequency adaptation (SFA) coefficient. D—3D graph presenting SFA coefficients (*Z*-axis), with *X*–*Y*-plane represented by normalized (to rheobase) current steps (a.u.) and instantaneous frequency (1/ISI, Hz) for DIV24 and DIV32 SCO neurons. E—AP regularity coefficient (a.u.) versus injected current with allometric function fitting (ax^b^, a and b are parameters, x—data points). F—SFA coefficient (a.u.) versus the injected current, with allometric function fitting (ax^b^, a and b are parameters, x—data points).

Analyses of AP kinetic properties (rise and decay time constants (τ); Supporting Information, Table S3) showed a pronounced difference in tau rise for DIV32 compared to DIV15, with a substantial decrease in tau rise occurring from DIV24 to DIV32 (Figure 2A). Tau decay decreased significantly from DIV15 to DIV32, indicating progressively faster repolarization. These changes in tau rise and tau decay indicate faster AP kinetics, consistent with neuronal maturation.

### Afterhyperpolarization (AHP) Properties

AHP amplitudes did not change significantly at the different culture time points, whereas AHP full width at the half-maximum (fwhm) significantly increased in the group DIV24 compared to DIV15 (Figure 2B). Evaluation of the kinetic properties of the AHP showed no significant changes (Figure 2B and Table S3).

### AP Firing Patterns

We analyzed AP frequency-current curves (f-I curves, Figure S1B), instantaneous frequencies (calculated from averaged interspike intervals, ISI), spike frequency adaptation (SFA) coefficients (the pattern of AP firing with an increase of interspike intervals over time), and regularity indices (Figure 2C–E). Analyses of regularity and SFA coefficients for neurons at DIV24 (*n* = 17) and DIV32 (*n* = 11), indicated early AP firing patterns with SFA (Figure 2D, F). Although such sequences were episodic (nonregular) and therefore could not be referred to as well-established patterns at this time point, they were observed in both groups. The majority of AP firing patterns showed high irregularity (as demonstrated by regularity indices).

### Immunohistochemical Characterization

To study molecular aspects of neuronal differentiation, we performed immunohistochemistry on sections from DIV24 and DIV32 SCOs using antibodies to specific neural and neuronal proteins, paired as follows (TUJ1/ISLET1, MAP2/GFAP, GFAP/NF, NeuN/Hb9, PAX2/ISLET1, PAX2/TUJ1, and Lhx1/5/MAP2; Table S4).

Staining with antibodies to the general neuron-specific protein β-III tubulin (TUJ1) revealed dense neuronal somata throughout SCO sections at both DIV24 and DIV32 (Figure 3A), indicating the presence of differentiating neurons. When combined with anti-ISLET1, a marker for immature motor neurons, the presence of motor neurons was confirmed in both age groups (Figure 3A, Supporting Information, Figure S2B).

**Figure 3:**
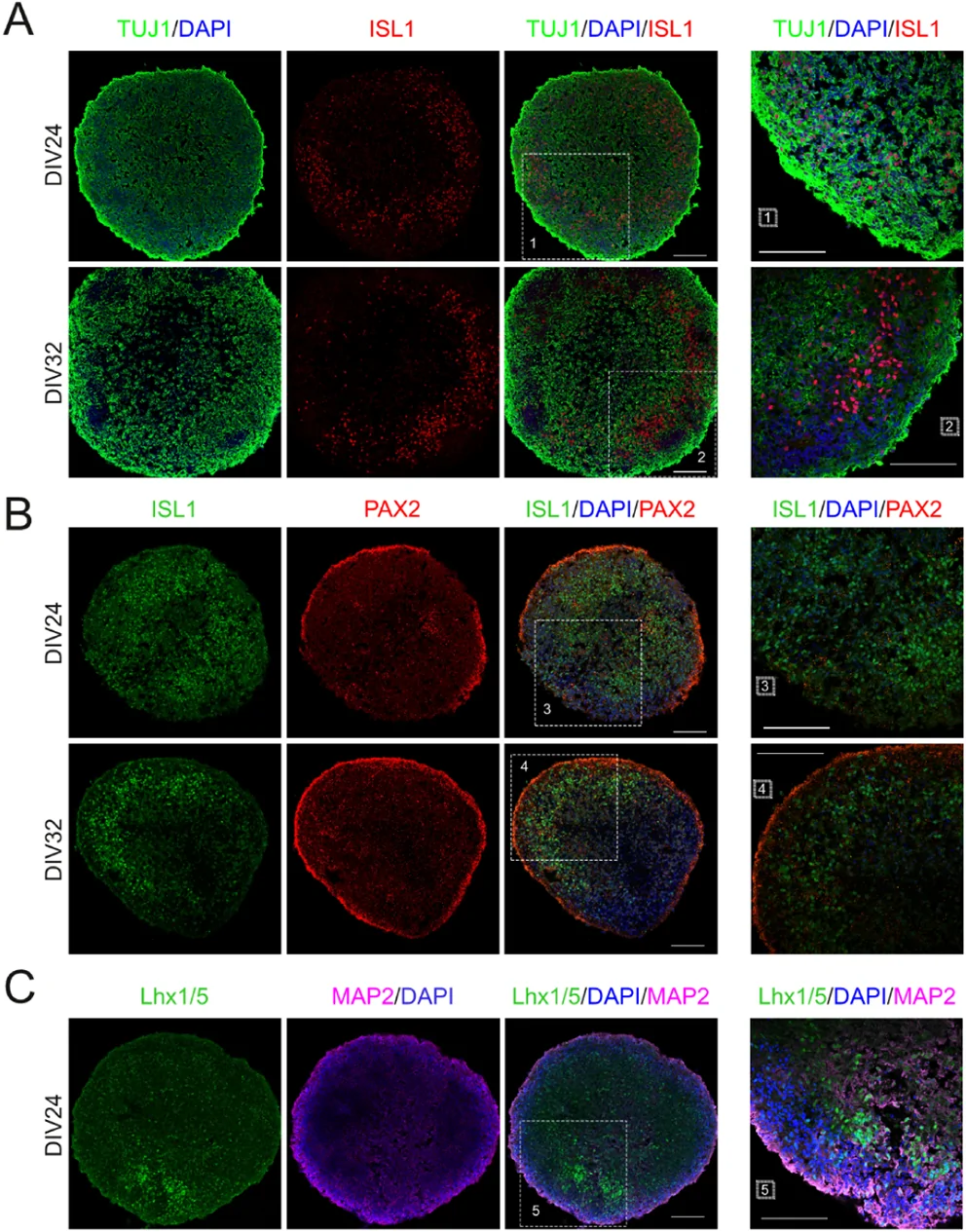
Expression of markers for immature motor- and interneurons in spinal cord organoids A—Representative images showing the coexpression of TUJ1 and ISLET1, merged with DAPI staining, in SCO at DIV24 (upper panels) and DIV32 (lower panels). B—Representative images of costaining for PAX2 with ISLET1 in DIV24 (upper panel) and DIV32 (lower panel) SCOs. C—Representative image of staining with LHX1/5 combined with MAP2, showing the pattern of LHX1/5-positive cell distribution in DIV24 SCOs. Scale bar: 100 μm.

Colabeling for PAX2 and ISLET1 yielded a distribution pattern of ISLET1-positive neurons in patches or clusters, similar to that observed with TUJ1/ISLET1 staining, and PAX2 labeling was detected in both DIV24 and DIV32 organoids (Figure 3B).

LHX1 and LHX5, which are coexpressed in multiple interneuron subtypes in the developing spinal cord—including early-born dI4 and dI6 inhibitory interneurons, as well as late-born inhibitory dILA neurons^26^—were assessed to track interneuron distribution. As shown in Figure 3C, large clusters of LHX1/5-positive cells were observed in DIV24 organoids, whereas no detectable fluorescent signal was present in DIV32 organoids (data not shown).

Staining for microtubule-associated protein 2 (MAP2) in combination with HB9 (a marker for postmitotic motor neurons, Figure 4A) and costaining for NeuN (a marker for mature neurons) and HB9 revealed a subset of HB9-positive neurons, indicating that the organoids at both time points already contain semimature motor neurons (Figure 4B, Supporting Information, Figure S1).

**Figure 4:**
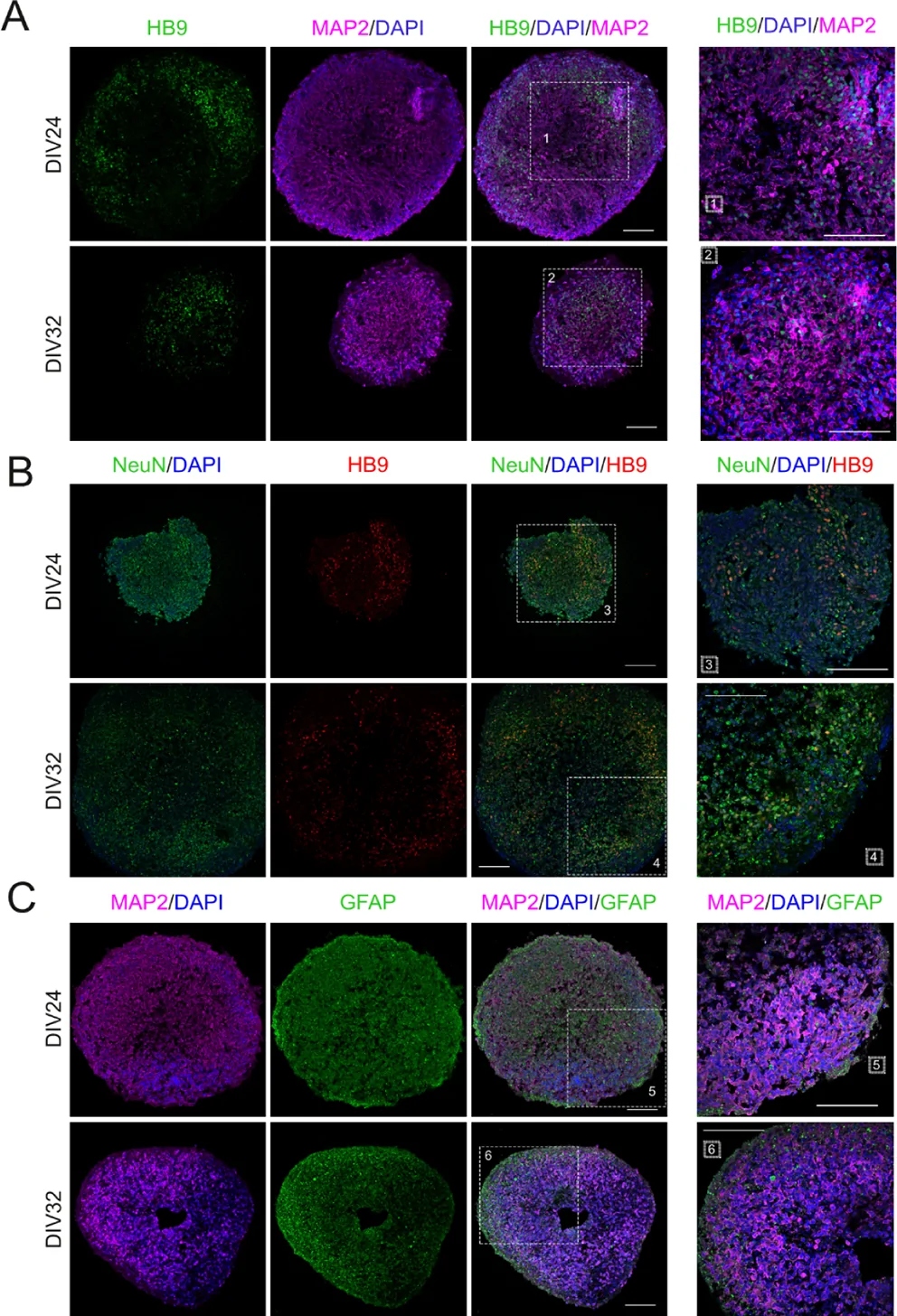
Expression of markers for postmitotic motor neurons and glial cells in DIV24/DIV32 spinal cord organoids. A—Representative confocal immunofluorescence images of SCO sections showing double-staining for HB9 markers and MAP2 (combined with DAPI) in DIV24 and DIV32 SCOs. B—Representative confocal immunofluorescence images of SCO sections showing double-staining for NeuN and HB9 markers (combined with DAPI) in DIV24 and DIV32 SCOs. C—Fluorescence images showing staining for MAP2 and GFAP in SCOs. GFAP-positive cells were detected at both time points. Scale bar: 100 μm.

Staining for MAP2, in combination with glial fibrillary acidic protein (GFAP), showed a dense distribution of developing neurons with sparse GFAP expression at both time points (Figure 4C). Similarly, staining with Neurofilament Heavy (NFH), combined with GFAP, produced comparable GFAP labeling patterns (Supporting Information, Figure S2). These findings are consistent with previous studies reporting traceable levels of glial elements at later differentiation stages (DIV50-DIV60).^21,27^

On the basis of immunohistochemical analysis, we conclude that the DIV24 and DIV32 spinal cord organoids contain multiple neuronal elements, including immature interneuron populations, early-developing glial cells, and immature as well as postmitotic motor neurons, with the latter representing the most abundant cell cluster.

Because of the high cellular density and significant spatial complexity of these 3D constructs, this histological characterization served to complement our primary investigation, which centered on the detailed electrophysiological assessment of functional neuronal profiles, providing a molecular context for the maturation states we observed.

## Discussion

The use of hiPSC-derived SCOs provides a significant advance in investigating the early functional maturation of human spinal neurons within a preserved 3D microenvironment. Given that all neuronal cells in hiPSC-derived SCOs must, by definition, be excitable, functional assessments have previously been performed using optical recording of calcium transients^19,28^ or extracellular recordings with 2D/3D MEA systems,^22^ which have covered only late maturation periods.^29,30^ While MEA is designed for the simultaneous recording of spiking activity from groups of neurons, it does not describe single-neuron performance or neuronal phenotype. Prior to our study, patch-clamp experiments had been reported only in replated neurons dissociated from spinal SCOs.^9,19^

Here, we provide analyses of whole-cell electrical recordings made from individual neurons with predefined morphologies within intact whole organoids, preserving 3D architecture and integral cellular microcircuitry. In the present work, we describe the principal characteristics and excitability profile of hiPSC-derived SCO neurons at the early stages of development. We identified progressive transitional changes in neuron membrane properties and excitability states from 2 weeks of differentiation onward, which could be defined as a critical period for neuronal maturation in SCOs.

First, we analyzed the passive membrane properties of the recorded neurons in three SCO differentiation stages. We found that resting membrane potential (RMP) values were significantly different in SCO neurons from DIV32 compared to those of DIV15, and we noted a tendency toward more hyperpolarized RMP over time in culture. Such changes in membrane properties have been previously reported for developing human EMS-derived motor neurons and are considered maturation-associated alterations.^31^ Both capacitance and cell time constant differed significantly in the pairwise comparison of the DIV15 and DIV24 groups, suggesting changes to membrane properties.

Further, we compared the voltage-gated ion channel activity of neurons in SCOs from the aforementioned age groups. Inward currents recorded in DIV15 SCOs were characterized by relatively small amplitudes and late activation, while the amplitude range for DIV24 SCOs was significantly shifted toward larger amplitudes. The current–voltage relationship curves (I–V) for DIV24 and DIV32 showed peak current activation at more negative potentials. The maximum current density was highest at DIV24. Collectively, we demonstrated substantial changes in inward (sodium) current activation threshold, amplitude, and I–V curves over time in culture, suggesting a switch in subtype and a dynamic increase in the number of active Na^+^ channels.

A central aspect of neuronal communication is the ability to propagate signals along the axon toward other neurons’ axon terminals, while the encoding of information relies on the generation of APs (spikes). The ability of neurons to generate APs either spontaneously or upon current injection in vitro is an important characteristic of functional postmitotic neurons. It is determined by the expression level and appropriate clustering of sodium and potassium channels on the axon initial segment and cell soma and is indicative of mature neurons.^32–35^ In our work, AP activation was successfully recorded in the majority of examined cells. We also observed an increased capacity to generate more complex waveforms and repetitive firing with time in culture. Neurons from DIV15 predominantly fired initial-type APs (23%) or single APs (61%), whereas DIV24 and DIV32 neurons tended to fire multiple APs, with ratios of 44% and 53%, respectively (Figure 1D). Analyses of AP parameters indicate a difference in AP amplitudes between the DIV15 and DIV24 groups.

Since the driving force of ion flux across the membrane is determined by the relative intra- and extracellular ion concentrations (i.e., the ion gradient), the AP dynamics apparently depend on subtle fluctuations in ion concentrations. The decrease in intracellular sodium concentrations results in higher peak voltages of APs in more mature neurons.^36^ APs at the early neuronal development stage are characterized by slow kinetics. More rapid depolarization and a shorter AP duration during maturation are driven by increased sodium and potassium channel expression, respectively.^37^ We found that parameters of AP activation (tau rise) differed significantly in DIV32 compared to DIV15, showing a progressive decrease in AP depolarization time in DIV24 SCOs. The AP half-width, known to decrease with neuronal maturation—making the AP narrower—was not substantially altered in either of the recorded groups, which could be indicative of a transitional stage rather than a fully mature state of neurons.

The encoding of information in neuronal networks is predetermined by the specific firing fashion, approximated to neuronal identity, as shown for regular-spiking neurons,^38^ fast-spiking interneurons,^39^ and intrinsic-bursting or neurons with adapting firing. The emergence of definitive firing patterns indicates the activation of signaling pathways toward the completion of neuronal functional identity.

Spike frequency adaptation (SFA) as a firing pattern is defined as the slowing of the firing rate over tens of seconds during sustained or intermittent current injection, i.e., an increase in interspike intervals.^40–42^ SFA in spinal MNs is thought to contribute to the optimal development of sustained muscle contractions and is a characteristic of MNs in different animal species^43,44^ and in hiPSC-derived MNs.^40–42,45^

Two of the previously proposed mechanisms for SFA cover an increase in outward current flowing through calcium-activated potassium channels and an increasing outward current produced by the electrogenic sodium–potassium pump. Additionally, the involvement of medium AHP conductance (apamin), BK-type Ca^2+^-dependent K^+^ channels (iberiotoxin), voltage-activated Ca^2+^ channels (CdCl_2_), M-current (linopirdine), and persistent Na^+^ currents (riluzole) was tested in multiple experiments,^45,46^ and fast-inactivating Na^+^ channels have also been suggested to be involved.^47^

Our quantitative analyses of AP firing patterns revealed the emergence of SFA in neurons from hiPSC-derived SCOs at least as early as DIV24, whereas most neurons showed highly irregular APs. Further study of SFA in human SCOs at later time points could contribute to a better understanding of neuronal identity and the underlying mechanisms in human MNs.

Our immunohistochemical analyses provide evidence of multiple neuronal types in SCOs at DIV24 and DIV32. The consistent expression of ISLET1 confirmed the presence of immature MNs, and the emergence of HB9 expression indicated a transition toward a more mature motor neuron phenotype. Expression of LHX1/5^26^ and PAX2 also indicates the presence of smaller populations of different spinal interneuron types. The sparse expression of GFAP suggests that glial maturation is limited at these stages.^21,27^

The electrical properties of spinal cord neurons are well documented in neonatal rats^48^ and mice,^49–51^ while only a few studies are available on spinal neuron maturation in human fetal spinal cord^52^ and human spinal neuron cultures.^53^ Maturation-related changes in the electrical properties of motor neurons have been reported for human embryonic stem cell-derived spinal motor neurons in culture,^31^ although there is insufficient information on the neuronal profile of 3D spinal cord organoid neurons. Here, we have described the principal characteristics and excitability profiles of hiPSC-derived SCO neurons in the early stages of development. We identified progressive transitional changes in neuron membrane properties and excitability states during DIV15-DIV32 culture and showed that this resembles the process of functional neuronal maturation described in rodent and human spinal cords.

Together with our immunohistochemical data, our findings indicate the presence of diverse neuron types as the source of the electrophysiological characteristics observed, reinforcing the notion that the SCO model can recapitulate early spinal cord development.

## Conclusions

Our study demonstrates that hiPSC-derived spinal cord organoids (SCOs) recapitulate the key electrophysiological features of early human spinal cord development. The SCO neurons exhibit progressive maturation, as evidenced by hyperpolarized resting membrane potentials, increased inward voltage-dependent current amplitudes, refined AP kinetics, and the early emergence of specific firing modes. In particular, we show that the SFA phenomenon appears at early stages of neuronal development, as observed in some SCO neurons. Furthermore, the abundant expression of motor-neuron-specific markers supports the physiological relevance of this model. Overall, our findings establish hiPSC-derived SCOs as robust in vitro platforms for investigating human spinal cord development and for advancing translational research in CNS disorders and cell replacement therapies.

## Materials and Methods

### Ethical Use of Animals or Human Participants in Research

The study utilized the human iPSC line HPSI0114i-vabj_3 (Wellcome Trust Sanger Institute, UK). No new human participants or samples were recruited for this study. The human iPSC line HPSI0114i-vabj_3 was obtained from the Wellcome Trust Sanger Institute/ECACC under a materials transfer agreement for academic, noncommercial research use. The original derivation and distribution of this cell line were performed under ethical approvals and donor consent arrangements applicable to the HipSci/Sanger resource. Because this study used an established anonymized cell line and did not involve the collection of new human samples, no additional human subjects’ approval or informed consent was required for the present experiments.

### Human Induced Pluripotent Stem Cell (hiPSC) Maintenance

Human iPSCs (HPSI0114i-vabj_3, Wellcome Trust Sanger Institute, Cambridgeshire, U.K.) were maintained in feeder-free conditions using StemFlex Medium (ThermoFisher, A3349401) on 6-well plates coated with 10 μg/cm^2^ Cultrex ECM (Bio-Techne Ltd., 3434–010–02). The medium was refreshed every other day. Cells at 70–80% confluency were passaged using 0.5 mM EDTA in PBS at a 1:5 ratio and reseeded. Cultures exhibiting no signs of spontaneous differentiation and high viability were used for spinal cord organoid differentiation.

### Spinal Cord Organoid Generation

hiPSC cultures (70–80% confluence) at about passage 10 were dissociated into single cells using Accutase (ThermoFisher, A1110501) and reaggregated into embryoid bodies in ultralow attachment U-bottom 96-well plates (Corning, CLS7007) at a seeding density of 10,000 cells per well in 150 μL of N2B27 medium supplemented with 10% knockout serum replacement (KSR; ThermoFisher, 10828010). Cultures were maintained in this medium from days in vitro (DIV) 0 to 15 (see below for a description of additional key reagents). N2B27 differentiation medium consisted of a 1:1 mixture of DMEM/F-12 and Neurobasal medium (ThermoFisher) containing 0.5% (v/v) N2 Supplement (ThermoFisher, 17502001), 1% (v/v) B27 Supplement without Vitamin A (ThermoFisher, 12587010), 1% MEM-NEAA, 1% GlutaMAX, 0.1 mM 2-mercaptoethanol, 0.5 μM ascorbic acid, and 1% penicillin–streptomycin (ThermoFisher, 15070063).

Key small molecules and growth factors were added to N2B27 medium as follows at the indicated DIV: DIV0–3:10 μM Y-27632 (AdooQ, A11001), 3 μM CHIR99021 (Peprotech, 2520691), and 20 ng/mL bFGF (peprotech, 100–18B). DIV0–6:10 μM SB431542 (Biogems, 3014193). DIV3–15:100 nM RA (peprotech, 3027949). DIV15–24:100 nM RA, and 15 ng/mL BMP4. DIV15–32:10 ng/mL BDNF (Peprotech, 450–02), and 20 ng/mL GDNF (Peprotech, 450–10). Medium changes were performed every 2 days.

### Immunohistochemistry

SCOs were harvested at DIV24 and DIV32, fixed in 4% PFA in PBS (pH 7.4) overnight at 4 °C, transferred to 30% sucrose in PBS for 24 h, embedded in OCT compound, and stored at −80 °C. The SCOs were sectioned at 10 μm using a cryostat.

For immunohistochemistry, sections were washed with PBS and blocked in TBS containing 10% goat serum and 0.2% Triton X-100 for 2 h at room temperature. Primary antibodies were applied overnight at 4 °C. After rinsing with TBS containing 1% goat serum, sections were incubated with appropriate secondary antibodies for 2 h at room temperature. Detailed antibody specifications and dilutions are provided in Table S4. Nuclei were counterstained with DAPI, and coverslips were mounted in a 50% PBS/glycerol solution.

Images were acquired using a Zeiss LSM700 confocal microscope at the Norwegian Core Facility for Human Pluripotent Stem Cells and processed with Zen Lite software. For quantitative analyses, 4–5 organoids at DIV24 and 3–4 organoids at DIV32 were assessed, unless otherwise stated.

### Electrophysiology

All electrophysiology experiments were performed at the Laboratory for Neural Development and Optical Recording (NDEVOR). SCOs were transferred from culture wells to a recording chamber containing room-temperature artificial cerebrospinal fluid (ACSF) in mM: 150 NaCl, 5 KCl, 11 glucose, 1 MgSO_4_, 2 CaCl_2_, 10 HEPES; pH 7.4; 310–320 mOsm/L. The internal pipette solution contained (in mM): 117.5 K-gluconate, 17.5 KCl, 10 HEPES, 0.2 EGTA, 5 Mg-ATP, 0.5 Na_3_GTP, 10 NaPhosphocreatine phosphate; pH 7.4; 300–310 mOsm/L. Patch electrodes were fabricated from borosilicate glass capillaries with an outer diameter of 1.5 mm (Sutter Instruments) using an automated puller (PC-10; Narishige).

Whole-cell patch-clamp recordings were performed in neurons from intact SCOs with diameter of 200 to 600 μm at DIV15, DIV24, and DIV32. Voltage-clamp mode was used to record inward and outward currents, mediated by voltage-gated Na^+^ and K^+^ channels, and current-clamp mode was used to assess passive membrane properties and to record rest membrane potentials (RMPs) and action potentials (APs).

Recordings were performed using a Multi-Clamp 700B amplifier (Molecular Devices). Signals were typically low-pass filtered with a corner frequency (−3 dB) of 5–6 kHz and sampled at 8–9 kHz using a DigiData 1322A (Molecular Devices). Amplifier management and data acquisition were performed under WinWCP software control (University of Strathclyde). Data analyses were performed with p-CLAMP 10 (Molecular Devices), OriginLab 8 (OriginLab Corp.), and custom-written Python scripts (python.org; JupyterLab, jupyter.org).

### Neuronal Morphology Reconstruction

Neurons were visually identified with an upright infrared differential interference contrast (IR-DIC) microscope (Olympus BX51WI) and captured with a CoolSNAP EZ with SSD ICX285 (Photometrics) video camera.

The fluorescent dye Alexa Fluor 488 was routinely added to the pipette solution at a concentration of ≈20 μM during patch-clamp recordings. DIC images of recorded neurons were captured at the beginning of each experiment. Fluorescence imaging was performed under TIRF microscopy (Olympus BX51WI). Fluorescence excitation was at 482(±20) nm using a 40X water-immersion objective (numerical aperture 0.8, Olympus), and a xenon light source (Sutter Instruments) with shutter control (VCM-D1, Uniblitz, Vincent Associates).

The images were acquired with a 0.9 s exposure time. Image analysis was conducted using Fiji/ImageJ software (http://fiji.sc)^54^ and GIMP (https://www.gnu.org). We collected and processed fluorescence images (2D or 3D) from 106 of 134 recorded neurons. A 3D reconstruction of neuronal morphology was performed for 32 neurons.

### Quantification and Statistical Analysis

Results are presented as means ± standard error of the mean (SEM), unless otherwise indicated. We have applied posthoc Bonferroni test for pairwise comparisons after performing a One-Way ANOVA test to identify which specific group means differ, controlling for Type I error inflation. Statistical significance was set at *p* < 0.05, with significance levels indicated by asterisks as follows: *p ≤ 0.05, **p < 0.01, ***p < 0.001. All analyses were performed using OriginLab 8 (OriginLab Corp.) and custom-made Python scripts.

## Supplementary Material



## Notes

Supplementary figures and tables show membrane properties of neurons in SCOs, schematic representations of AP and AHP analysis, AP activation versus stimuli, and additional immunohistochemistry experiments (PDF)
