# Supplementary material for: Human iPSC-Derived Spinal Cord Organoids – The First Electrophysiological Assessment of Neuronal Maturation
Source: ACS Chem Neurosci. 2026 Jul 28;17(16):3048–57. doi: 10.1021/acschemneuro.5c00823 (PMC13495673; doi:10.1021/acschemneuro.5c00823)
Supplement: Supplementary file 1 [file cn5c00823_si_001.pdf]

Supporting information for Publication

Human iPSC-derived spinal cord organoids – the first electrophysiological assessment of neuronal maturation

Oleksandr Ievglevskyi[1, 2], Denis Zosen[3], Jordi Requena Osete[1,4], Joel C. Glover[5,6,] Ragnhild E. Paulsen[3], Srdjan Djurovic[1,4], Elena Kondratskaya[1,4]*

[1]Department of Medical Genetics, Oslo University Hospital, Oslo, 0450, Norway.

[2]Neurobiology, SISSA, Trieste, 34136; Computational and System Neuroscience, ISAS, Camerino, 62032, Italy.

[3]Section for Pharmacology and Pharmaceutical Biosciences, Department of Pharmacy, University of Oslo, 0371, Norway.

[4]Centre for Precision Psychiatry, Division of Mental Health and Addiction, University of Oslo, and Oslo University Hospital Oslo, 0450, Norway.

[5]Laboratory of Neural Development and Optical Recording (NDEVOR), Division of Physiology, Department of Molecular Medicine, Institute of Basic Medical Sciences, University of Oslo, 0372, Norway.

[6]Norwegian Center for Stem Cell Research, Institute of Immunology and Transfusion Medicine, Oslo University Hospital, Oslo, 0372, Norway.

*corresponding author.

Present affiliation: Department of Clinical Medicine, University of Bergen, Bergen, 5020, Norway. olena.kondratska@uib.no

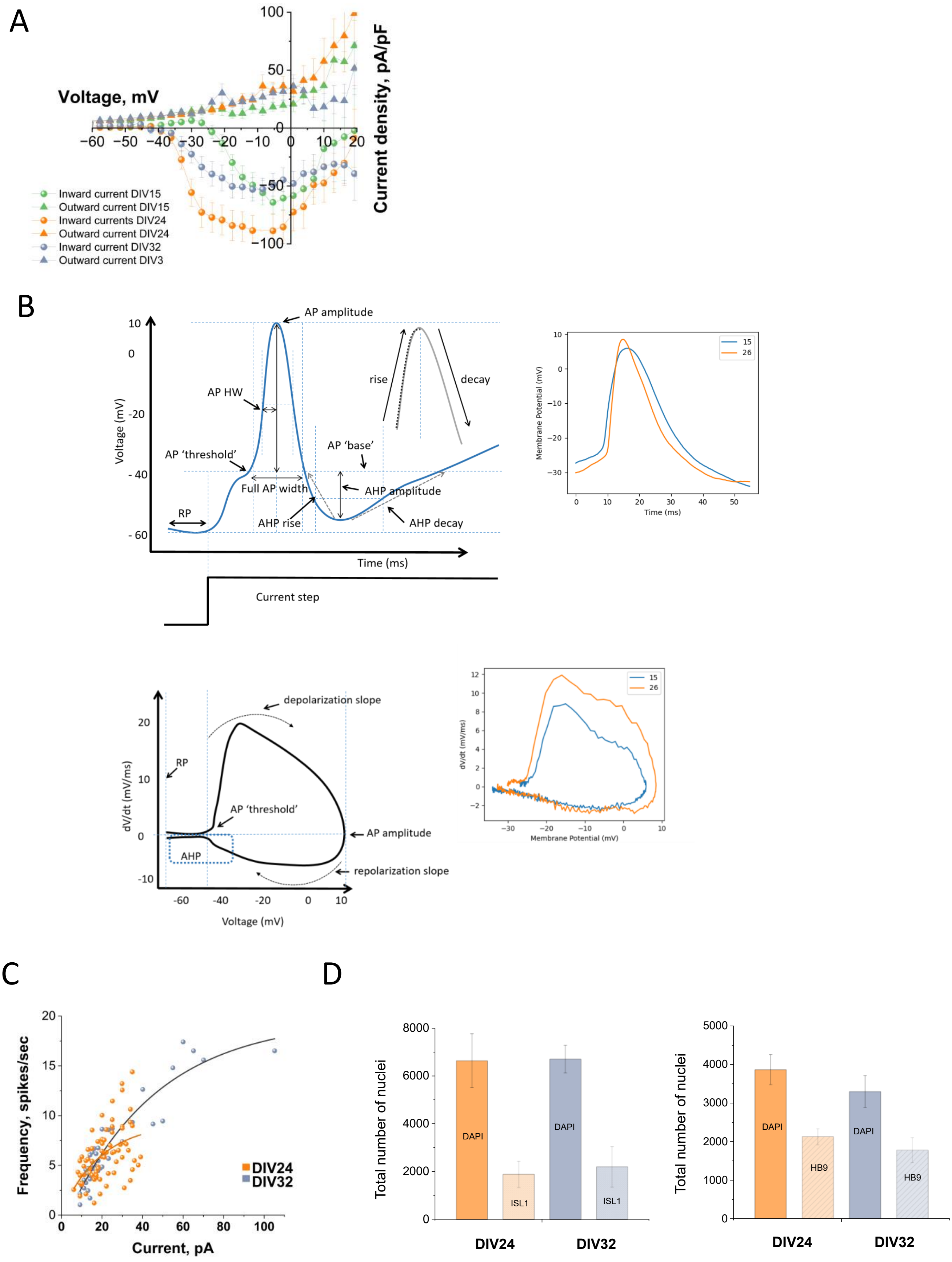
A
Voltage, mV
Current density, pA/pF
Inward current DIV15
Outward current DIV15
Inward currents DIV24
Outward current DIV24
Inward current DIV32
Outward current DIV3
B
AP amplitude
AP HW
AP 'threshold'
Full AP width
AHP rise
AP 'base'
AHP amplitude
AHP decay
RP
rise
decay
Voltage (mV)
Time (ms)
Current step
Membrane Potential (mV)
depolarization slope
repolarization slope
AHP
dV/dt (mV/ms)
Voltage (mV)
C
Frequency, spikes/sec
Current, pA
DIV24
DIV32
D
Total number of nuclei
DAPI
ISL1
HB9
DIV24
DIV32

Figure S1.

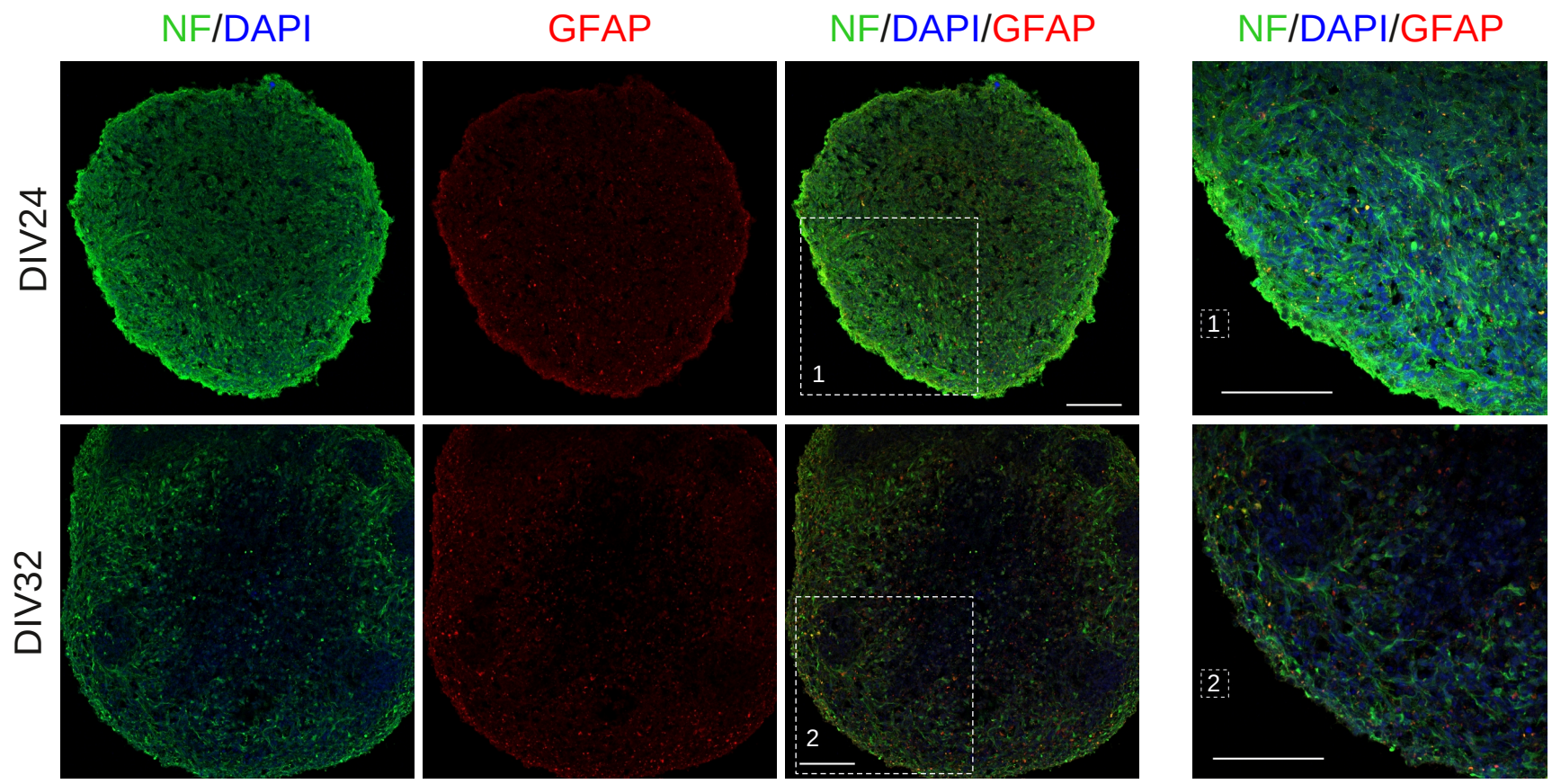

Figure S2

FIGURE S1.

A – Representative average IV curves for inward and outward currents densities (pA/pF vs. V) recorded from neurons in DIV15, DIV24, and DIV32.

B – upper panel – Graphical plot of experimentally evaluated selected AP parameter in immature SCO neurons.

Lower panel – Graphical plot of AP profile evaluation showing the voltage derivative (dV/dt) versus voltage (V) used in AP analyses.

C – Summary graph representing the frequency-to-injected current ratio for AP trains recorded in DIV24 and DIV32 neurons via exponential fit curves.

D – Quantification of neurons expressing ISL1 and HB9 in groups DIV24 and DIV32.

FIGURE S2.

Representative image of double staining for NF-H and GFAP in DIV24 (upper panel) and DIV32 organoids (lower panel). Scale bar corresponds to 100 μm.

Table S1. Passive neuronal properties (N corresponds to number of individual recordings)

Parameter	Group	N	Mean	SE
Capacitance (pF)	DIV15	15	195.36	8.52
	DIV24	42	288.87	10.66
	DIV32	23	232.67	16.16
Rest membrane potential (mV)	DIV15	15	-60.28	3.04
	DIV24	42	-67.54	1.04
	DIV32	23	-70.73	2.8
Membrane resistance (MΩ)	DIV15	15	160.45	29.95
	DIV24	42	170.084	10.7
	DIV32	23	200.81	34.74
Cell time constant (ms)	DIV15	15	37.15	6.56
	DIV24	42	70.21	5.81
	DIV32	23	59.35	9.71
Input resistance (MΩ)	DIV15	15	177.13	32.44
	DIV24	42	189.73	11.91
	DIV32	23	222.28	40.75

Table S2. Inward current (I_{inward}) parameters

Parameter	Group	N	Mean	SE
Voltage threshold (mV)	DIV15	22	-13.79	3.18
	DIV24	57	-22.34	1.23
	DIV32	30	-17.82	1.85
Current peak amplitude (pA)	DIV15	22	-235	36.94
	DIV24	57	-417.61	48.08
	DIV32	30	-245.27	30.39
Current density, I_{max}/C (pA/pF)	DIV15	22	-30.7	4.62
	DIV24	57	-48.12	5.75
	DIV32	30	-29.33	3.74

Table S3. Action potential (AP) and afterhyperpolarization (AHP) parameters

Parameter	Group	N	Mean	SE
AP Amplitude (mV)	DIV15	10	26.32	3.06
	DIV24	39	26.46	1.36
	DIV32	17	15.8	1.76
AP HWHM (ms)	DIV15	10	5.83	0.86
	DIV24	39	5.59	0.31
	DIV32	17	6.07	0.43
Rise tau (ms)	DIV15	10	8.85	2.17
	DIV24	39	5.82	0.53
	DIV32	17	4.48	0.6
Decay tau (ms)	DIV15	10	2.12	0.33
	DIV24	39	1.96	0.2
	DIV32	17	1.15	0.1
AHP amplitude (mV)	DIV15	10	7.06	1.25
	DIV24	39	11.27	0.92
	DIV32	17	10.88	1.17
AHP tau rise (ms)	DIV15	10	10.29	2.56
	DIV24	39	13.14	0.74
	DIV32	17	13.28	1.66
AHP tau decay (ms)	DIV15	10	24.33	4.88
	DIV24	39	15.86	2.02
	DIV32	17	12.32	3.23
AHP FWHM (ms)	DIV15	10	12.73	2.28
	DIV24	39	20.77	1.22
	DIV32	17	18.08	2.67

Table S4. List of primary and secondary antibodies used for immunohistofluorescence

Antibody	Species	Concentration/dilution	Company	Reference
GFAP (Glial Fibrillary Acidic Protein)	rabbit	1:10 000	Dako	Z0334
MAP2	chicken	1:10 000	Abcam	AB5392
VGlut1	rabbit	1: 500	Synaptic Systems	135302
Anti-HB9 HB9/HLXB9/MNX1	rabbit	1: 500	Abcam	AB221884
Anti-Islet 1	rabbit	1:500	Abcam EP4182	AB109517
Anti-Islet1	mouse	1:250	DSHB	39.4D5
Anti-NeuN	mouse	1:300	Millipore	MAB377 3519381
LHX1/5	mouse	1:500	DSHB	A115338
Anti-NeuN	rabbit	1:300	Abcam	Ab177487
Anti- Tuj1 (TubIII)	mouse	1:500	R&D system	MAB1195-SP
GAD65/67	rabbit	1:400	Millipore Sigma	ABN904
Neurofilament Heavy	chicken	1:1000	Biolegend	822601
PAX2	rabbit	1:200	Abcam	A38738
Alexa 488	Goat anti chicken	1:400	Invitrogen	A11039
Alexa 555	Goat anti rabbit	1:500	Invitrogen	2775963
Alexa 488	Goat anti mouse	1:500	Jackson	115-545-062
Alexa 488	Goat anti rabbit	1:500	Invitrogen	1855440
Alexa 647	Goat anti chicken	1:500	Invitrogen	A21449
